# Global Asthma Network Phase I Surveillance: Geographical Coverage and Response Rates

**DOI:** 10.3390/jcm9113688

**Published:** 2020-11-17

**Authors:** Philippa Ellwood, Eamon Ellwood, Charlotte Rutter, Virginia Perez-Fernandez, Eva Morales, Luis García-Marcos, Neil Pearce, M Innes Asher, David Strachan, on behalf of the GAN Phase I Study Group

**Affiliations:** 1 Department of Paediatrics: Child and Youth Health, Faculty of Medical and Health Sciences, University of Auckland, 1023 Auckland, New Zealand; e.ellwood@auckland.ac.nz (E.E.); i.asher@auckland.ac.nz (M.I.A.); 2 Department of Medical Statistics, London School of Hygiene and Tropical Medicine, London WC1E 7HT, UK; Charlotte.Rutter1@lshtm.ac.uk (C.R.); Neil.Pearce@lshtm.ac.uk (N.P.); 3 Pediatric Allergy and Pulmonology Units, ‘Virgen de la Arrixaca’ University Children’s Hospital, University of Murcia, ARADyAL network and Biomedical Research Institute of Murcia (IMIB-Arrixaca), 30394 Murcia, Spain; virperez@um.es (V.P.-F.); lgmarcos@um.es (L.G.-M.); 4 Biomedical Research Institute of Murcia (IMIB-Arrixaca) and Department of Public Health Sciences, University of Murcia, 30394 Murcia, Spain; embarto@hotmail.com; 5 Centre for Global NCDs, London School of Hygiene and Tropical Medicine, London WC1E 7HT, UK; 6 Population Health Research Institute, St George’s University of London, London SW17 ORE, UK; d.strachan@sgul.ac.uk

**Keywords:** global, asthma, surveillance, responses, children, adults’ epidemiology

## Abstract

Background—The Global Asthma Network (GAN) Phase I is surveying school pupils in high-income and low- or middle-income countries using the International Study of Asthma and Allergies in Childhood (ISAAC) methodology. Methods—Cross-sectional surveys of participants in two age groups in randomly selected schools within each centre (2015–2020). The compulsory age group is 13–14 years (adolescents), optionally including parents or guardians. Six to seven years (children) and their parents are also optional. Adolescents completed questionnaires at school, and took home adult questionnaires for parent/guardian completion. Children took home questionnaires for parent/guardian completion about the child and also adult questionnaires. Questions related to symptoms and risk factors for asthma and allergy, asthma management, school/work absence and hospitalisation. Results—53 centres in 20 countries completed quality checks by 31 May 2020. These included 21 centres that previously participated in ISAAC. There were 132,748 adolescents (average response rate 88.8%), 91,802 children (average response rate 79.1%), and 177,622 adults, with >97% answering risk factor questions and >98% answering questions on asthma management, school/work absence and hospitalisation. Conclusion—The high response rates achieved in ISAAC have generally been maintained in GAN. GAN Phase I surveys, partially overlapping with ISAAC centres, will allow within-centre analyses of time-trends in prevalence.

## 1. Introduction

The Global Asthma Network (GAN) [1,2] was formed in 2012 as a joint initiative by members of the International Study of Asthma and Allergies in Childhood (ISAAC) and the International Union Against Tuberculosis and Lung Disease, following their co-production of the first Global Asthma Report (GAR), launched in 2011 at the time of the United Nations high-level meeting on non-communicable diseases [3]. Estimates of the worldwide burden of asthma in that report were based very largely on the ISAAC Phase III surveys (2001–2003) of 13–14-year-olds (adolescents) and 6–7-year-olds (children) [4], with time trends from centres which also participated in ISAAC Phase I (1994–1995) [5], plus the World Health Surveys of adults (2002–2003) [6]. The need for updated surveillance of asthma prevalence, severity, diagnosis and management, highlighted in GAR 2011 [3], has become more pressing since then [7], as very few studies anywhere in the world have evaluated trends in asthma prevalence and related risk factors over the last decade [8].

GAN Phase I was developed to address this information gap, with these hypotheses:

(1) Globally, the burden of asthma is changing in adults and children;
(2) There is large variation in the diagnosis of asthma;
(3) In many locations, asthma is under-diagnosed and its management is suboptimal; and
(4) There are potentially modifiable risk factors for asthma.

Its aims were:

(1) To conduct asthma surveillance around the world in two age groups of school pupils, and their parents, measuring prevalence, severity, management and risk factors, following the methods of ISAAC Phase III;
(2) To examine time trends in prevalence, severity, management and risk factors from centres which completed ISAAC Phase III; and
(3) To evaluate the appropriateness of asthma management, especially access to quality-assured essential asthma medicines, as defined by WHO [9].

Although modelled closely on the study design and methodology of ISAAC Phase III, GAN Phase I has extended its scope to include adults, for whom there are limited global data on asthma prevalence [8], severity and risk factors, and to assess asthma management, which is commonly suboptimal in low-income settings [7]. This paper summarises the progress of GAN Phase I at 31 May 2020, when the dataset was temporarily frozen for the first round of analyses including centres which completed the quality checks by this date.

## 2. Methods

GAN has collaborators from 383 centres in 137 countries all of whom answered the call for an Expression of Interest (EOI). Of the EOIs, 136 centres in 58 countries registered to participate in GAN Phase I. Of these registered centres some have completed GAN Phase I and provided data to this study, while some, because of timing, will be included in later publications. Other centres have been unable to undertake Phase I at all because of unforeseen circumstances. Many centres in each of these categories have contributed to other published GAN surveys [10,11,12,13].

GAN Phase I is a cross-sectional, multi-centre, multi-country study undertaken between 2015–2020. Its methodology has been described and justified elsewhere [2] and detailed in an online manual [14]. Each centre was required to obtain approval from their local ethics committee prior to the start of their study. 

Briefly, each GAN centre is based on a defined geographical area, within which a minimum of 10 schools were selected at random (or all schools, if less than ten). All students of a specified age within these schools were studied, selected by grade/level/year, or by chronological age. The sample size estimates of 1000–3000 are stringent because of the number of hypotheses being tested, and high response rates are sought. As in ISAAC, two age groups of school pupils participated: adolescents and children. Centres that undertook ISAAC Phase III and/or ISAAC Phase I were expected to use the same study design and sampling frame in GAN. As in ISAAC Phase III, translations into the local language were required and centres followed the ISAAC protocol for translation, back translation to English, and comparison between the two [15].

The compulsory age group was adolescents, who self-completed written questionnaires at school. Additionally, in some centres, the ISAAC international video questionnaire showing different scenes of asthma in children of a variety of ethnicities was shown [16]. A self-completed risk factor questionnaire, developed for ISAAC Phase III, was strongly recommended in this age group. In ISAAC surveys, there was no contact with the parents of the adolescent age group, but for GAN, it was recommended (but not essential) that the parents/guardians of the adolescents were also surveyed. 

This optional parental questionnaire obtained information on the prevalence of asthma, rhinitis and eczema symptoms among adults, plus questions on asthma management and risk factors. The adult symptoms questionnaire combined items from ISAAC and the European Community Respiratory Health Survey (ECRHS) [17] to cover the range of chest symptoms and diagnoses that might be related to asthma in young and middle-aged adults.

The inclusion of children was optional, as with ISAAC Phase III, who took written questionnaires home to be completed by their parents. These included the ISAAC questionnaire on the child’s symptoms used in Phases I and III, and the risk factor questionnaire used in Phase III. In GAN it was recommended (but not essential) to add the parental questionnaire to ascertain the prevalence of asthma, rhinitis and eczema symptoms among adults in the household.

Data from each centre were submitted to the GAN Global Centre (Auckland, New Zealand) together with a descriptive centre report. Following initial quality control checks in Auckland, the data were transferred to one of two designated GAN Phase I data centres for checking and analysis: Murcia (Spain) for Spanish- and Portuguese-speaking centres, and London (United Kingdom), for centres using all other languages. A harmonised approach to data processing, checking and analysis was developed, using Stata versions 13–15.

Estimation of participation rates among children and adolescents followed the conventions previously adopted in ISAAC Phase III. High levels of participation are sought as it is a concern that absent school pupils may be away from school due to symptoms of asthma, rhinitis or eczema. A participation rate of at least 80% for the adolescents and 70% for the children is desirable [2,14]. The denominator was the number of pupils in the cluster sample and the numerator was the number of core symptom questionnaires returned with at least some symptom data. 

We were unable to calculate a conventional response rate for the adults as it was not known how many adults received questionnaires (because some schoolchildren have only one parent or guardian). Therefore, a “per child” approach was taken to estimate adult response rate, as follows. The denominator was the number of school-aged respondents (index schoolchildren) to whom one or more adult questionnaires were distributed. The numerator was the number of index schoolchildren for whom one or more adult questionnaires were returned. For centres which distributed adult questionnaires to both age groups of schoolchildren, the numerators and denominators were combined to derive a single estimate of “per child” adult response rate.

It was not possible to derive this measure of adult response rate for three centres (Costa Rica (whole country study), Guatemala City, Guatemala; Tegucigalpa, Honduras) where adult responses were not linked to the child identifier.

## 3. Results

**Figure 1:**
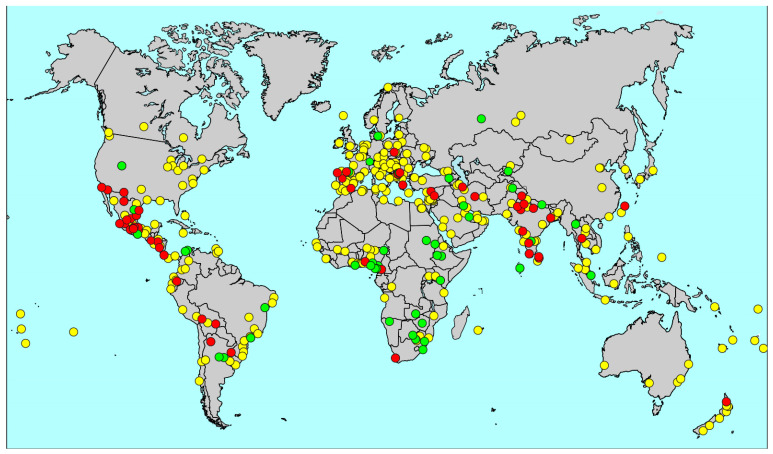
Global Asthma Network (GAN) Centres. Centres registered with GAN identifying those that completed data collection before end of May 2020 (red); registered centres expected to complete GAN Phase I later (green); centres collaborating with GAN but not expected to contribute Phase I data (yellow).

By 31 May 2020, 53 centres in 20 countries had submitted and completed quality checks of data and methodology. Figure 1 shows the location of these centres, also 84 centres in 38 countries which formally registered an intention to complete GAN Phase I but were unable to do so, and the remaining GAN collaborating centres. Most centres completed their fieldwork before the onset of the COVID-19 pandemic, but surveys were still active in Iran (Yazd and Karaj) and Greece (Athens) in spring 2020, where fieldwork was truncated due to school closures in the pandemic lockdown. 

**Figure 2:**
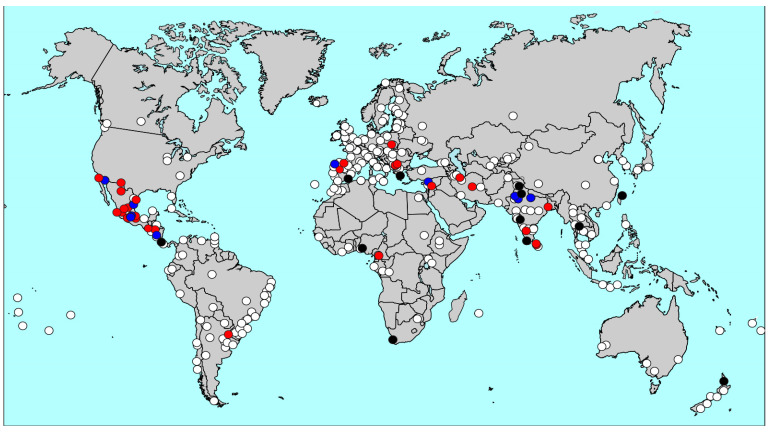
Overlap of GAN Phase I and ISAAC Centres. Centres that completed GAN Phase I checks before 31 May 2020 (red); GAN Phase I centres included in ISAAC Phase III but not ISAAC Phase I (blue); GAN Phase I centres included in ISAAC Phase I and ISAAC Phase III (black*); ISAAC Phase III only centres (white). * Athens, Greece contributed data to GAN and ISAAC Phase I, but not ISAAC Phase III.

Twenty-one of the 53 centres had previously participated in ISAAC Phase III (including 17 contributing data on both age groups) and 12 had previously participated in ISAAC Phase I (including 9 with data on both age groups). All of the 12 ISAAC Phase I centres except for Athens also participated in ISAAC Phase III. The geographical overlap between ISAAC and GAN centres is shown in Figure 2. Forty of the 53 GAN centres also contributed data on adult symptoms, risk factors and disease management, as summarised in Table 1.

Table 2 summarises the numbers of pupils and number of schools for which responses were received in GAN Phase I, by age group and questionnaire section (symptoms, risk factors, management and morbidity). When deriving the number of valid responses to the asthma management and asthma-related morbidity questions, respondents who legitimately skipped these sections because they had answered negatively to asthma symptoms were included in the count of responders. 

Overall, responses were received for 132,748 adolescents attending 1260 schools (with risk factor information on 99.3% and management/morbidity information on 98.8%) and 91,802 children attending 1506 schools (with risk factor information on 99.3% and management/morbidity information on 99.5%). Additionally, there were responses for 177,622 adults, with risk factor information on 97.7% and 98.2% providing information on asthma management, work absence, or hospitalisation. These 177,622 adults relate to 100,011 school pupils that returned adult questionnaires, comprising 50,416 adolescents and 49,595 children.

The stringent response criteria were able to be met by 45 (85%) of the 53 GAN Phase I centres for adolescents, 33 (80%) of the 41 GAN Phase I centres for children and by 24 (65%) of the 37 GAN Phase I centres for adults. Lower rates in some centres occurred due to schools closing because of the COVID-19 pandemic. Table 3 compares the response rates for the core symptom questionnaires by age group for each GAN Phase I centre and the corresponding response rates in earlier ISAAC surveys, where relevant. Across all GAN centres, the mean participation rate was 88.8% for adolescents and 79.1% for children (compared to 88.0% and 84.5%, respectively, in ISAAC Phase III). For GAN Phase I centres which were also ISAAC Phase III centres, mean response rates were 90.0% for adolescents and 79.0% for children in GAN compared with 89.3% and 84.4%, respectively, in ISAAC Phase III. One or more responses to the adult symptom questionnaire were received from an average of 73.2% of households contacted.

## 4. Discussion

GAN Phase I has completed fieldwork with data and methodology quality checks in a large number of centres in both high-income and low- or middle-income countries including representation from all inhabited continents. This broad geographical coverage is expected to expand as a number of centres have commenced fieldwork but not yet submitted completed data. However, four countries (India, Kosovo, Mexico and Spain) account for two-thirds of the datasets received by 31 May 2020 which may limit the international generalisability of the findings. 

Overlap between ISAAC and GAN is less extensive than anticipated, but 21 diverse centres will provide local time-trends in disease prevalence. These within-centre trends can be used, with caution, to inform projections of trends in prevalence among the remaining centres in ISAAC Phase III, which offer a much more widespread international representation than has been achieved so far in GAN.

Careful checks of the methodology used (centre report and data checks), as with ISAAC, ensured clarity on how the study was actually done and any variations encountered. The high levels of responses achieved in ISAAC have generally been maintained in GAN, suggesting that estimates of prevalence and severity of asthma will be representative of the populations surveyed. Sample sizes in most centres achieved the recommended target of 3000 children per age group, leading to precise estimates of disease prevalence, but in a few centres the numbers of respondents are substantially lower (Table 2).

The response rate in both age groups in Guatemala was unusually low (Table 3) and we explored the possible reasons for this. In both age groups, questionnaires were sent home for completion by the parents, whereas in other centres, the adolescents self-completed the questionnaires in class. This probably explains the exceptionally low response rate among 13–14-year-olds in Guatemala.

Extension of ISAAC methodology to include questions about parental symptoms was an attempt to fill gaps in knowledge about the prevalence, severity, diagnosis and management of asthma and related risk factors among young and middle-aged adults. Parents of schoolchildren are not a random or representative sample of the adult population, but the high response rates achieved in many of the study centres suggest that useful results could be obtained in this manner. The total number of adult respondents in GAN (177,622) is comparable with two previous international studies of young and middle-aged adults, discussed below. 

The ECRHS, (1991–1993) recruited 137,619 participants aged 20–44 years in 48 centres in 22 countries (including 5 non-European countries: Algeria, Australia, India, New Zealand, USA) [17]. The GAN adult questionnaire incorporates core ECRHS items, but the geographical overlap with ECRHS countries is limited. The World Health Survey (WHS, 2002–2003) interviewed 178,215 adults aged 18–45 years from 70 countries and included a few questions about asthma and related symptoms among a general health questionnaire [6]. Although there is better geographical overlap with GAN, at least at the country level, the WHS questionnaire lacks detail which limits the scope for historical comparisons with GAN data on asthma severity.

Among adolescents and children, ISAAC offered a global perspective on time trends in asthma prevalence from the mid-1990s to the early 2000s [5,18] but very few ISAAC centres have repeated their local surveys subsequently, prior to GAN. In Brazil, adolescents in Curitiba, Recife and São Paulo were studied in ISAAC Phases I (1994) and III (2003) and again in 2012 [19] and in South Santiago, Chile, ISAAC Phases I and III were completed, and a further survey of asthma in adolescents completed in 2015 [20]. Three GAN Phase I studies with previous ISAAC data have been published: in Bangkok, Thailand, [21] and four Mexican centres [22,23]. Time trends in these centres have been summarised elsewhere [8].

With the closure of this first round of data in GAN Phase I, these temporal and geographical comparisons can now be extended to a wider and more diverse range of study centres. These results will form the basis of analyses for journal publications in the near future. However, GAN centres that were unable to meet the criteria for this first data compilation can still contribute results to future analyses and publications. The GAN Phase I Study Group is listed at the Appendix A.

In summary, GAN Phase I offers, for the first time in nearly two decades, new standardised worldwide data on prevalence and severity of asthma in adolescents, children and adults. This will enable comparisons to be made over time, and contribute a new picture of the global burden of asthma, rhinoconjunctivitis and eczema. Not only will risk factors be examined, but also time trends in these, and global variation, shedding light on causation. The methodology which ISAAC started has a proven track record of over nearly 30 years, and now extends to adults (parents) as well as adolescents and children. The high response rates achieved in a range of settings are testimony to the feasibility of the approach and give confidence in the estimates obtained.

## Figures and Tables

**Table 1:** Number of study centres contributing data for each GAN Phase I module and age-group, with corresponding data for International Study of Asthma and Allergies in Childhood (ISAAC) Phases I and III.

	GAN Phase I Centres			ISAAC Phase III Centres *		ISAAC Phase I Centres *	
Questionnaire Module	6–7	13–14	Adults	6–7	13–14	6–7	13–14
Symptoms:							
Asthma (written)	41	53	40	144 {17}	233 {21}	91 {9}	155 {12}
Asthma (video)	NA	29	NA	NA	139 {8}	NA	99 {3}
Rhinoconjunctivitis	41	53	NA	144 {17}	233 {21}	91 {9}	155 {12}
Eczema	41	53	40	142 {17}	231 {21}	91 {9}	155 {12}
Risk factors:							
ISAAC Phase 3 questions	40	52	38	75 {17}	122 {21}	ND	ND
Active smoking	NA	52	38	ND	ND	ND	ND
Perinatal questions	39	NA	NA	ND	ND	ND	ND
Indoor environment	39	NA	38	ND	ND	ND	ND
Asthma-related:							
Management (now)	41	53	40	ND	ND	ND	ND
Management (infancy)	39	NA	NA	ND	ND	ND	ND
School absence	41	53	40	ND	ND	ND	ND
Work absence	NA	NA	40	ND	ND	ND	ND
Hospitalisation	41	53	40	ND	ND	ND	ND

* Numbers of centres also participating in GAN Phase I in parentheses. NA Not applicable (module not included for that age group). ND No data (module not included in ISAAC data collection).

**Table 2:** Number of participants (P) and number of schools (S) responding to each GAN module by study centre and age-group.

	6–7-Year-Olds						13–14-Year-Olds						Adults					
	Symptoms		Risk Factors		Management		Symptoms		Risk Factors		Management		Symptoms		Risk Factors		Management	
Centre Name	P	*S*	P	*S*	P	*S*	P	*S*	P	*S*	P	*S*	P	*S*	P	*S*	P	*S*
Yaounde	722	*27*	722	*27*	703	*27*	1066	*22*	1066	*22*	1056	*22*	860	*32*	832	*32*	824	*32*
Ibadan	0	*0*	0	*0*	0	*0*	2897	*23*	2894	*23*	2810	*23*	2321	*23*	2321	*23*	2217	*23*
Cape Town	0	*0*	0	*0*	0	*0*	3979	*29*	3976	*29*	3879	*29*	0	*0*	0	*0*	0	*0*
Taipei	3036	*25*	3036	*25*	3034	*25*	3474	*24*	3465	*24*	3464	*24*	9689	*49*	9673	*49*	9594	*49*
Bangkok	3067	*7*	3067	*7*	3063	*7*	3206	*6*	3201	*6*	3084	*6*	5418	*13*	5416	*13*	5311	*13*
Yazd	0	*0*	0	*0*	0	*0*	5141	*48*	5141	*48*	5141	*48*	0	*0*	0	*0*	0	*0*
Karaj	572	*39*	0	*0*	572	*39*	754	*42*	0	*0*	754	*42*	1175	*75*	0	*0*	1175	*75*
Lattakia	1115	*9*	1078	*9*	1111	*9*	1215	*10*	1214	*10*	1203	*10*	0	*0*	0	*0*	0	*0*
Damascus	0	*0*	0	*0*	0	*0*	1100	*11*	1100	*11*	1100	*11*	0	*0*	0	*0*	0	*0*
Kottayam	2099	*50*	2099	*50*	2085	*50*	2090	*20*	2088	*20*	2050	*20*	6940	*69*	6937	*69*	6743	*69*
New Delhi	2516	*54*	2516	*54*	2516	*54*	3024	*59*	3024	*59*	3024	*59*	9449	*113*	9449	*113*	9010	*113*
Chandigarh	2473	*57*	2473	*57*	2473	*57*	3000	*54*	3000	*54*	2999	*54*	10,386	*111*	10,386	*111*	10,384	*111*
Bikaner	2600	*45*	2600	*45*	2551	*45*	2702	*33*	2702	*33*	2702	*33*	10,495	*78*	10,495	*78*	10,473	*78*
Jaipur	2296	*44*	2296	*44*	2250	*44*	3060	*57*	3057	*57*	2977	*57*	8933	*101*	8902	*101*	8524	*101*
iLucknow	2969	*32*	2969	*32*	2931	*32*	2968	*31*	2969	*31*	2933	*31*	11,820	*63*	11,786	*63*	11,405	*63*
Kolkata	0	*0*	0	*0*	0	*0*	2998	*37*	2998	*37*	2886	*37*	7823	*91*	7818	*91*	7547	*91*
Pune	2404	*26*	2404	*26*	2403	*26*	3030	*34*	3030	*34*	3021	*34*	8000	*60*	7994	*60*	7909	*60*
Mysuru (Mysore)	2730	*30*	2730	*30*	2730	*30*	3051	*29*	3051	*29*	3051	*29*	11,178	*59*	11,178	*59*	11,177	*59*
Peradeniya	1492	*12*	1492	*12*	1455	*12*	1696	*11*	1696	*11*	1547	*11*	0	*0*	0	*0*	0	*0*
Anuradhapura	2180	*10*	2180	*10*	2120	*10*	2986	*10*	2989	*10*	2638	*10*	0	*0*	0	*0*	0	*0*
Uruguaiana	0	*0*	0	*0*	0	*0*	1058	*17*	1052	*17*	1057	*17*	896	*17*	896	*17*	884	*17*
Costa Rica	1936	*34*	1936	*34*	1936	*34*	1338	*33*	1338	*33*	1316	*33*	3272	*67*	3272	*67*	3102	*67*
Guatemala City	1072	*39*	1072	*39*	1071	*39*	1420	*42*	1408	*42*	1400	*42*	1078	*30*	1078	*30*	1055	*29*
Tegucigalpa	361	*22*	361	*22*	359	*22*	1431	*65*	1431	*65*	1415	*65*	254	*10*	254	*10*	252	*10*
Mexico City North	2515	*58*	2515	*58*	2498	*58*	3375	*9*	3370	*9*	3375	*9*	5231	*66*	5219	*66*	5104	*66*
Guadalajara	2082	*21*	2082	*21*	2075	*21*	2519	*13*	2518	*13*	2516	*13*	489	*20*	487	*20*	483	*20*
Mexicali	2001	*37*	2001	*37*	1999	*37*	2479	*20*	2464	*20*	2469	*20*	2436	*41*	2427	*41*	2427	*41*
Ciudad Victoria	2444	*20*	2444	*20*	2439	*20*	2468	*8*	2465	*8*	2467	*8*	6239	*28*	6202	*28*	6149	*28*
San Luis Potosí	2108	*28*	2108	*28*	2108	*28*	2580	*19*	2580	*19*	2579	*19*	2835	*27*	2833	*27*	2801	*27*
Tijuana	2082	*47*	2082	*47*	2072	*47*	2601	*13*	2595	*13*	2577	*13*	1397	*26*	1395	*26*	1376	*26*
Toluca Urban	2712	*21*	2712	*21*	2702	*21*	2650	*6*	2642	*6*	2643	*6*	6162	*27*	6122	*27*	6072	*27*
Toluca Rural	2975	*17*	2976	*17*	2974	*17*	3122	*16*	3114	*16*	3091	*16*	7587	*33*	7583	*33*	7470	*33*
Chihuahua	1969	*33*	1969	*33*	1962	*33*	2180	*7*	2103	*7*	2161	*7*	0	*0*	0	*0*	0	*0*
Ciudad Juárez	2117	*39*	2118	*39*	2114	*39*	2443	*16*	2439	*16*	2426	*16*	2610	*37*	2598	*37*	2601	*37*
Michoácan	2166	*39*	2166	*39*	2156	*39*	2504	*14*	2502	*14*	2503	*14*	2232	*39*	2232	*39*	2206	*39*
Xalapa	3716	*83*	3717	*83*	3712	*83*	3339	*21*	3335	*21*	3327	*21*	0	*0*	0	*0*	0	*0*
Còrdoba	2746	*60*	2746	*60*	2738	*60*	2991	*25*	2980	*25*	2989	*25*	2839	*35*	2832	*35*	2829	*35*
Puerto Vallarta	2241	*46*	2241	*46*	2238	*46*	2439	*15*	2439	*15*	2428	*15*	0	*0*	0	*0*	0	*0*
Aguascalientes	3175	*19*	3176	*19*	3165	*19*	3336	*19*	3334	*19*	3331	*19*	2907	*33*	2898	*33*	2861	*33*
Matamoros	806	*24*	806	*24*	799	*24*	2892	*12*	2882	*12*	2865	*12*	1315	*24*	1306	*24*	1298	*24*
Managua	3162	*59*	3162	*59*	3127	*59*	3131	*50*	3126	*50*	2973	*50*	0	*0*	0	*0*	0	*0*
Prishtina	0	*0*	0	*0*	0	*0*	1054	*14*	1056	*14*	1052	*14*	2006	*14*	2006	*14*	1977	*14*
Gjakova	0	*0*	0	*0*	0	*0*	676	*5*	676	*5*	676	*5*	1352	*5*	1352	*5*	1350	*5*
Prizren	0	*0*	0	*0*	0	*0*	1427	*10*	1427	*10*	1427	*10*	2712	*10*	0	*0*	2699	*10*
Gjilan	0	*0*	0	*0*	0	*0*	1200	*6*	1200	*6*	1200	*6*	1835	*6*	1835	*6*	1834	*6*
Ferizaj	0	*0*	0	*0*	0	*0*	890	*9*	890	*9*	885	*9*	1371	*9*	1372	*9*	1328	*9*
Katowice	1462	*36*	1462	*36*	1460	*36*	3185	*29*	3184	*29*	3180	*29*	2220	*35*	2219	*35*	2201	*35*
Auckland	1538	*22*	1538	*22*	1538	*22*	1885	*7*	1885	*7*	1860	*7*	3002	*29*	2994	*29*	2986	*29*
Athens	0	*0*	0	*0*	0	*0*	1934	*20*	1934	*20*	1934	*20*	1897	*20*	1897	*20*	1897	*20*
Cartagena	3509	*61*	3509	*61*	3496	*61*	3436	*26*	3430	*26*	3428	*26*	6961	*60*	6956	*60*	6832	*60*
Salamanca	2388	*51*	2388	*51*	2387	*51*	3485	*31*	3484	*31*	3481	*31*	0	*0*	0	*0*	0	*0*
Cantabria	2841	*75*	2841	*75*	2836	*75*	4381	*47*	4372	*47*	4374	*47*	0	*0*	0	*0*	0	*0*
A Coruña	3407	*48*	3407	*48*	3407	*48*	3462	*26*	3461	*26*	3455	*26*	0	*0*	0	*0*	0	*0*
All centres	91,802	*1506*	91,197	*1467*	91,365	*1506*	13,2748	*1260*	131,777	*1218*	131,179	*1260*	177,622	*1685*	173,452	*1600*	174,367	*1684*

**Table 3:** Response rates for 6–7 and 13–14 year age groups to the written symptom questionnaires in GAN Phase I, ISAAC Phases I and III, by study centre and age-group. (The adult response rate was estimated on a “per child” basis *).

		GAN Phase I				ISAAC Phase III			ISAAC Phase I		
		Survey	Response (%)			Survey	Response (%)		Survey	Response (%)	
Country	Centre Name	Years	6–7	13–14	Adult *	Years	6–7	13–14	Years	6–7	13–14
Cameroon	Yaounde	2018–19	53.8	99.9	34.6 ^a^						
Nigeria	Ibadan	2018	-	85.0	79.5 ^c^	2001–02		99.7	1995		76.4
South Africa	Cape Town	2017	-	84.4	^d^	2002		83.4	1995		82.8
Taiwan	Taipei	2016–17	76.3	93.0	84.5 ^a^	2001–02	96.8	95.9	1995	92.2	93.2
Thailand	Bangkok	2017–18	86.3	97.9	86.1 ^a^	2001	72.8	93.8	1995–96	90.8	74.8
Iran	Yazd	2020	-	71.3	^d^						
Iran	Karaj	2019–20	72.0	71.9	88.6 ^a^						
Syrian Arab Republic	Lattakia	2019	93.0	99.6	^d^	2001–03	99.1	99.8			
Syrian Arab Republic	Damascus	2018	-	91.7	^d^						
India	Kottayam	2017–18	68.4	85.3	97.5 ^a^	2001–03	96.4	98.5	1994–95	78.1	90.7
India	New Delhi	2017–18	80.9	100.0	85.7 ^a^	2001–02	82.4	86.7	1994–95	99.2	100
India	Chandigarh	2017–18	100.0	100.0	95.5 ^a^	2001–02		99.4	1994–95	94.0	97.4
India	Bikaner	2017–18	86.7	90.1	99.8 ^a^	2001		95.4			
India	Jaipur	2017–18	75.8	98.7	84.4 ^a^	2001	75.4	87.4			
India	Lucknow	2017	91.3	94.0	99.7 ^a^	2001–02	85.7	75.0			
India	Kolkata	2017–18	-	99.9	80.2 ^c^						
India	Pune	2017–18	79.8	99.6	81.4 ^a^	2001–02	90.4	70.8	1994–95	99.6	99.8
India	Mysuru (Mysore)	2017–18	90.9	99.5	97.4 ^a^						
Sri Lanka	Peradeniya	2018	74.6	80.8	^d^						
Sri Lanka	Anuradhapura	2018	72.7	85.4	^d^						
Brazil	Uruguaiana	2016–18	-	88.2	76.7 ^c^						
Costa Rica	Costa Rica	2017–18	64.5	66.9	^e^	2001–02	80.9	69.6	1994–95	84.1	91.4
Guatemala	Guatemala City	2018	32.2	40.6	^e^						
Honduras	Tegucigalpa	2017–18	76.5	98.0	^e^						
Mexico	Mexico City North	2015–16	86.7	93.8	55.9 ^a^	2002–03	91.6	99.8			
Mexico	Guadalajara	2016	83.3	90.0	12.1 ^b^						
Mexico	Mexicali	2015–16	77.0	83.7	32.7 ^a^	2002–03	74.3	93.6			
Mexico	Ciudad Victoria	2015–16	81.5	82.3	78.6 ^a^	2003	73.1	79.5			
Mexico	San Luis Potosí	2015–16	99.4	97.3	36.7 ^a^						
Mexico	Tijuana	2015–16	83.3	86.7	41.4 ^b^						
Mexico	Toluca Urban	2015–16	95.7	98.1	65.5 ^a^						
Mexico	Toluca Rural	2015–16	93.0	94.6	69.1 ^a^	2002	89.5	86.1			
Mexico	Chihuahua	2015–16	87.5	87.2	^d^						
Mexico	Ciudad Juárez	2016–17	84.7	88.8	36.7 ^a^						
Mexico	Michoacán	2016	90.3	92.7	75.8 ^b^						
Mexico	Xalapa	2016–17	92.9	90.2	^d^						
Mexico	Còrdoba	2016	91.5	93.5	30.2 ^a^						
Mexico	Puerto Vallarta	2015–17	93.4	90.3	^d^						
Mexico	Aguascalientes	2015–16	90.7	95.3	44.0 ^a^						
Mexico	Matamoros	2015–17	80.6	93.3	93.7 ^b^						
Nicaragua	Managua	2018	87.9	90.5	^d^	2002	96.0	94.5			
Kosovo	Prishtina	2017	-	99.9	99.9 ^c^						
Kosovo	Gjakova	2018	-	90.1	100.0 ^c^						
Kosovo	Prizren	2017	-	89.0	99.7 ^c^						
Kosovo	Gjilan	2017	-	80.0	81.5 ^c^						
Kosovo	Ferizaj	2017	-	99.9	85.1 ^c^						
Poland	Katowice	2017–18	36.8	79.1	85.6 ^b^						
New Zealand	Auckland	2018–19	63.7	85.5	51.3 ^a^	2001	84.6	92.3	1992–93	90.2	94.6
Greece	Athens	2020	-	75.5	99.9 ^c^				1994–95		87.0
Spain	Cartagena	2015–16	65.9	73.8	61.5 ^a^	2001–02	72.3	79.6	1993	68.5	95.1
Spain	Salamanca	2017–18	73.7	95.0	^d^						
Spain	Cantabria	2017–18	56.2	77.4	^d^						
Spain	A Coruña	2018–19	71.0	92.1	^d^	2003	73.8	93.6			

* Adult response rate per child, derived as the percentage of schoolchildren that had one or more adult questionnaires returned, combined across age groups when both age groups were studied: (a) both age groups; (b) 6–7-year-olds only; (c) 13–14-year-olds only; (d) neither age group; (e) adult responses not linked to child identifiers, so no response rate for adults can be derived.

## Appendix A

**GAN Phase I Study Group**

**Global Asthma Network Steering Group:** I Asher, University of Auckland, Auckland, New Zealand; N Billo, Joensuu, Finland; K Bissell, School of Population Health, University of Auckland, Auckland, New Zealand; Chiang C-Y, Division of Pulmonary Medicine, Department of Internal Medicine, Wan Fang Hospital, Taipei Medical University, Taipei, Taiwan; A El Sony, The Epidemiological Laboratory for Public Health and Research, Khartoum, Sudan; P Ellwood, University of Auckland, Auckland, New Zealand; L García-Marcos, Virgen de la Arrixaca University Children’s Hospital, Murcia, Spain; J Mallol, University of Santiago de Chile (USACH), Santiago, Chile; G Marks, University of New South Wales, Sydney, Australia; K Mortimer, Liverpool School of Tropical Medicine and Aintree University Hospital NHS Foundation Trust, Liverpool, United Kingdom; N Pearce, London School of Hygiene and Tropical Medicine, London, United Kingdom; D Strachan, St George’s, University of London, London, United Kingdom.

**Global Asthma Network International Data Centres: Auckland:** P Ellwood, E Ellwood, I Asher, University of Auckland, Auckland, New Zealand; **Murcia:** V Pérez-Fernández, E Morales, University of Murcia, Murcia, Spain; L García-Marcos, Pediatric Allergy and Pulmonology Units, ‘Virgen de la Arrixaca’ University Children’s Hospital, University of Murcia, ARADyAL network and Biomedical Research Institute of Murcia (IMIB-Arrixaca), Murcia, Spain; **London:** C Rutter, R Silverwood, S Robertson, Neil Pearce, London School of Hygiene & Tropical Medicine, London, United Kingdom; D Strachan, St George’s, University of London, London, United Kingdom.

**Global Asthma Network Principal Investigators:** Brazil: M Urrutia-Pereira, Federal University of Pampa, UNIPAMPA (Uruguaiana); Cameroon: AE Ndikum, The University of Yaounde 1 (Yaounde); Costa Rica: ME Soto-Quirós, University of Costa Rica (Costa Rica); Greece: K Douros, National and Kapodistrian University of Athens (Athens); Guatemala: L Pérez-Martini, Asociación Guatemalteca de Neumología y Cirugía de Tórax (Guatemala City); Honduras: SM Sosa Ferrari, Instituto Nacional Cardiopulmonar (Tegucigalpa); India: M Sabir, Maharaja Agrasen Medical College Agroha (Bikaner); M Singh, Postgraduate Institute of Medical Education and Research (Chandigarh); V Singh*, Asthma Bhawan (Jaipur); AG Ghoshal, National Allergy Asthma Bronchitis Institute (Kolkata (19)); TU Sukumaran, PIMS Thiruvalla (Kottayam); S Awasthi, King George’s Medical University (Lucknow); PA Mahesh, JSS University (Mysuru); SK Kabra, All India Institute of Medical Sciences (New Delhi); S Salvi, Chest Research Foundation (Pune); Iran: M Tavakol, Alborz University of Medical Sciences (Karaj); N Behniafard, Shahid Sadoughi University of Medical Sciences (Yazd); Kosovo: I Bucaliu-Ismajli, The principal center of family care (Ferizaj); L Pajaziti, (Gjakova); V Gashi, American Hospital in Kosovo (Gjilan); LN Ahmetaj*, University Hospital of Prishtina (Prishtina); V Zhjeqi, University of Prishtina (Prizren); Mexico: MG Sanchez Coronel, COMPEDIA (Colegio Mexicano de Pediatras (Aguascalientes); HL Moreno Gardea, Hospital Angeles Chihuahua (Chihuahua); G Ochoa-Lopez, Department of Pediatric Allergology (Ciudad Juárez); R García-Almaráz, Hospital Infantil de Tamaulipas (Ciudad Victoria); JA Sacre Hazouri, Instituto Privado de Alergia, (Córdoba); DD Hernández-Colín, Hospital Civil De Guadalajara Juan I Menchaca (Guadalajara); N Rodriguez-Perez, Instituto de Ciencias y Estudios Superiores de Tamaulipas (Matamoros); JV Mérida-Palacio, Centro de Investigacion de Enfermedades Alergicas y Respiratorias (Mexicali); BE Del Río Navarro*, Service of Allergy and Clinical immunology, Hospital Infantil de México (Mexico City North); LO Hernández-Mondragón, CRIT de Michoacán (Michoacán); Md Juan Pineda, Universidad de Guadalajara (Puerto Vallarta); Bd Ramos García, Instituto Mexicano del Seguro Social (San Luis Potosí); AJ Escalante-Dominguez, Hospital General Tijuana [Isesalud] (Tijuana); EM Navarrete-Rodriguez, Hospital Infantil de Mexico Federico Gomez (Toluca Urban); FJ Linares-Zapién, Centro De Enfermedades Alergicas Y Asma de Toluca (Toluca Rural); J Santos Lozano, Medica san Angel (Xalapa); New Zealand: I Asher, University of Auckland (Auckland); Nicaragua: JF Sánchez, Hospital Infantil Manuel de Jesús Rivera (Managua); Nigeria: AG Falade, University of Ibadan and University College Hospital (Ibadan); Poland: G Brożek, Medical University of Silesia (Katowice); South Africa: HJ Zar, SA MRC Unit on Child & Adolescent Health (Cape Town); Spain: A Bercedo Sanz, Cantabrian Health Service (Cantabria); L García-Marcos*, Pediatric Allergy and Pulmonology Units, ‘Virgen de la Arrixaca’ University Children’s Hospital, University of Murcia, ARADyAL network and Biomedical Research Institute of Murcia (IMIB-Arrixaca), Murcia, Spain (Cartagena); A López-Silvarrey Varela, Fundacion Maria Jose Jove (La Coruña); J Pellegrini Belinchon, Universidad de Salamanca (Salamanca); Sri Lanka: JC Ranasinghe, Teaching Hospital Peradeniya (Anuradhapura); ST Kudagammana, University of Peradeniya (Peradeniya); Syrian Arab Republic: G Alkhayer, Damascus Private University (Damascus); G Dib, Lattakia University (Lattakia 13–14); Y Mohammad*, National Center for research and training for chronic respiratory disease and co_morbidities (Lattakia 6–7); Taiwan: J-L Huang, Chang Gung University (Taipei); Thailand: P Vichyanond*, Mahidol University (Bangkok).

* National Coordinators

Global Asthma Network Adult Age Group Principal Investigators not named above:

Cameroon: GA Ajeagah, The University of Yaounde 1 (Yaounde); Costa Rica: M Soto-Martinez, University of Costa Rica (Costa Rica); Greece: K Priftis, National and Kapodistrian University of Athens (Athens); Guatemala: M Cohen-Todd, Asociacion Guatemalteca De Neumologia Y Cirugia De Torax (Guatemala City); Honduras: J Sanchez, Instituto Nacional Cardiopulmonar (Tegucigalpa); India: SK Kochar, Sardar Patel Medical College (Bikaner); N Singh, Asthma Bhawan (Jaipur); N Sit, National Allergy Asthma Bronchitis Institute (Kolkata (19)); S Sinha, All India Institute of Medical Sciences (New Delhi); M Barne, Chest Research Foundation (Pune); Kosovo: B Ajeti, The Principal center of Family Care (Ferizaj); LH Lleshi, (Gjakova); V Lokaj-Berisha, University of Prishtina (Prizren); Mexico: Md Ambriz-Moreno, (Matamoros); OJ Saucedo-Ramirez, Hospital Angeles Pedregal (Mexico City North); CA Jiménez González, Universidad Autonoma of San Luis Potosí (San Luis Potosí); Taiwan: K-W Yeh, (Taipei); Thailand: S Chinratanapisit, Bhumibol Adulyadej Hospital (Bangkok).

**Global Asthma Network National Co-ordinators not named above:** Brasil: D Solé, Escola Paulista de Medicina, Federal University of São Paulo, São Paulo.

**ISAAC Phase III Principal Investigators:** Costa Rica: ME Soto-Quirós*, University of Costa Rica (Costa Rica); India: M Sabir, Maharaja Agrasen Medical College Agroha (Bikaner); L Kumarⴕ, Department of Pediatrics (Chandigarh); V Singh, Asthma Bhawan (Jaipur); T Sukumaran, PIMS Thiruvalla (Kottayam); S Awasthi, King George’s Medical University (Lucknow); SK Sharma, All India Institute of Medical Sciences (New Delhi (7)); NM Hanumante, Ruby Hall Clinic (Pune); Mexico: R García-Almaráz, Hospital Infantil de Tamaulipas (Ciudad Victoria); JV Merida-Palacio, Centro de Investigacion de Enfermedades Alergicas y Respiratorias (Mexicali Valley); BE Del-Río-Navarro, Service of Allergy and Clinical immunology, Hospital Infantil de México (Ciudad de México (1)); FJ Linares-Zapién, Centro De Enfermedades Alergicas Y Asma de Toluca (Toluca); New Zealand: MI Asher*, University of Auckland (Auckland); Nicaragua: JF Sánchez*, Hospital Infantil Manuel de Jesús Rivera (Managua); Nigeria: BO Onadeko, (Ibadan); South Africa: HJ Zar*, University of Cape Town (Cape Town); Spain: L García-Marcos*, Pediatric Allergy and Pulmonology Units, ‘Virgen de la Arrixaca’ University Children’s Hospital, University of Murcia, ARADyAL network and Biomedical Research Institute of Murcia (IMIB-Arrixaca), Murcia, Spain (Cartagena); A López-Silvarrey Varela, Fundacion Maria Jose Jove (A Coruña); Syria: Y Mohammad, National Center for Research and Training in Chronic Respiratory Diseases—Tishreen University (Lattakia); Taiwan: J-L Huang*, Chang Gung University (Taipei); Thailand: P Vichyanond*, Mahidol University (Bangkok).

* National Coordinators

ⴕ Deceased

**ISAAC Phase III National Co-ordinators not named above:** Mexico: M Baeza-Bacab, University Autónoma de Yucatán, Yucatán; Syrian Arab Republic: S Mohammad, Tishreen University, Lattakia.

**ISAAC Phase I Principal Investigators:** Costa Rica: ME Soto-Quirós*, University of Costa Rica (Costa Rica); Greece: CH Gratziou*, National Kapodistrian University of Athens (Athens); India: L Kumar, Department of Pediatrics (Chandigarh); T Sukumaran, PIMS Thiruvalla (Kottayam); K Chopra, Maulana Azad Medical College (New Delhi (7)); NM Hanumante, Ruby Hall Clinic (Pune); New Zealand: MI Asher*, University of Auckland (Auckland); Nigeria: BO Onadeko, (Ibadan); Spain: L García-Marcos*, Pediatric Allergy and Pulmonology Units, ‘Virgen de la Arrixaca’ University Children’s Hospital, University of Murcia, ARADyAL network and Biomedical Research Institute of Murcia (IMIB-Arrixaca), Murcia, Spain (Cartagena); Taiwan: K-H Hsiehⴕ, Chang Gung Children’s Hospital (Taipei); Thailand: P Vichyanond*, Mahidol University (Bangkok); South Africa: R Erlich, University of Cape Town (Cape Town);

* National Coordinators

ⴕ Deceased

**ISAAC Phase I National Co-ordinators not named above:** India: J Shah, Jaslok Hospital & Research Centre, Mumbai.
